# Potential of the NKG2D/NKG2DL Axis in NK Cell-Mediated Clearance of the HIV-1 Reservoir

**DOI:** 10.3390/ijms20184490

**Published:** 2019-09-11

**Authors:** Maria G. Desimio, Daniela A. Covino, Margherita Doria

**Affiliations:** Academic Department of Pediatrics, Bambino Gesù Children’s Hospital, IRCCS, 00165 Rome, Italy; mariagiovanna.dsm@gmail.com (M.G.D.); covino.daniela@gmail.com (D.A.C.)

**Keywords:** HIV-1 eradication, natural killer (NK) cells, NKG2D, latency reversing agents, immunotherapy

## Abstract

Viral persistency in latently infected CD4^+^ T cells despite antiretroviral therapy (ART) represents a major drawback in the fight against HIV-1. Efforts to purge latent HIV-1 have been attempted using latency reversing agents (LRAs) that activate expression of the quiescent virus. However, initial trials have shown that immune responses of ART-treated patients are ineffective at clearing LRA-reactivated HIV-1 reservoirs, suggesting that an adjuvant immunotherapy is needed. Here we overview multiple lines of evidence indicating that natural killer (NK) cells have the potential to induce anti-HIV-1 responses relevant for virus eradication. In particular, we focus on the role of the NKG2D activating receptor that crucially enables NK cell-mediated killing of HIV-1-infected cells. We describe recent data indicating that LRAs can synergize with HIV-1 at upregulating ligands for NKG2D (NKG2DLs), hence sensitizing T cells that exit from viral latency for recognition and lysis by NK cells; in addition, we report in vivo and ex vivo data showing the potential benefits and drawbacks that LRAs may have on NKG2D expression and, more in general, on the cytotoxicity of NK cells. Finally, we discuss how the NKG2D/NKG2DLs axis can be exploited for the development of effective HIV-1 eradication strategies combining LRA-induced virus reactivation with recently optimized NK cell-based immunotherapies.

## 1. Introduction

Development of an efficacious antiretroviral therapy (ART) has dramatically decreased the morbidity and mortality of patients infected with human immunodeficiency virus type 1 (HIV-1). However, ART suppresses HIV-1 replication but does not eradicate the virus that persists in a replication-competent form in latently infected cellular reservoirs, mainly memory CD4^+^ T cells, where it is rapidly reactivated following treatment interruption. For this reason, HIV-1-infected patients need life-long ART administration, which can cause viral resistance and long-term toxicity; hence finding a cure for HIV-1 is still a major challenge for biomedical research.

Most research efforts towards a HIV-1 cure have been focused on the complete eradication of viral reservoirs. Progress in defining the molecular mechanisms of HIV-1 latency and in developing in vitro and in vivo latency models have allowed the development of HIV-1 reactivation and clearance strategies referred to as ‘shock and kill’ approaches [1]. The ‘shock’ consists of using latency reversing agents (LRAs) that induce expression of the quiescent provirus, which should lead to the killing of reactivated infected cells by the host immune system or virus-induced cytolysis, while the de novo produced virus is exposed to the effect of ART. In initial clinical trials, though, LRA administration increased cellular HIV-1 RNA but failed to decrease reservoir size [2,3], indicating that HIV-1 reactivation should be combined with therapies that stimulate the antiviral immune response. Indeed, various studies have demonstrated that cytotoxic CD8^+^ T cell (CTL) responses of ART-treated patients cannot efficiently clear infected cells that exit latency, a phenomenon that was linked to the scarcity or dysfunction of the patients’ HIV-1-specific CTLs [4,5] and to the presence of CTL escape mutations in latent viral genomes [6]. To boost the ‘killing’ phase, various immunological approaches are under development, including therapeutic HIV-1 vaccinations, antibody-based strategies, and immunomodulatory therapies [3,7,8].

Interestingly, recent studies have indicated that natural killer (NK) cells, one of the main types of effector cells of innate immunity, have an important role in the clearance of HIV-1 reservoirs, encouraging the exploration of NK cell-based approaches to achieve HIV-1 eradication.

NK cells possess a natural cytotoxic activity against virus-infected cells and tumor cells that is independent of prior antigen encounters, hence not affected by CTL escape mutants, and is regulated through the balance of opposing signals delivered by activating and inhibitory receptors [9]. Under normal conditions, NK cells are kept in a ‘off’ state through the interaction of inhibitory receptors (e.g., inhibitory Killer-cell Immunoglobulin-like Receptors-iKIRs-and NKG2A) with their cognate ligands belonging to the human leukocyte antigen class I (HLA-I) family of molecules expressed on the membrane of healthy cells. Upon tumor transformation or viral infection, cells lose, in part or completely, HLA-I expression, thus evading CTL recognition, and upregulate/express de novo ligands for activating NK cell receptors (i.e., NKp30, NKp44, NKp46—referred to as Natural Cytotoxicity Receptors or NCRs, NKG2D, activating KIRs—aKIRs, NKG2C, DNAM-1), hence becoming targets for the killing activity of NK cells in which triggering signals have prevailed. NK cells can also kill antibody-coated targets via antibody-dependent cellular cytotoxicity (ADCC) triggered by the Fcγ receptor CD16 recognizing the constant region of IgG antibodies; moreover, NK cells regulate immune responses via the production of various cytokines and chemokines as well as by a complex network of interactions with other cell types [10,11]. Various studies based on immunogenetic correlations, clinical, and experimental evidence have clearly demonstrated that NK cells play an important role in the anti-HIV-1 immune response [12]. Although existence of antigen-specific NK cells has not been demonstrated in humans, evidence indicating that NK cells can exert an HIV-1-specific pressure in vivo has been provided [13]. Recently, a transcriptome-wide study on HIV-1-infected patients showed that a novel pathway involving the recycling endosome effector protein RAB11Fip5 has an impact on NK cell functionality and is associated with the production of broadly neutralizing antibodies (bNabs), bringing into focus the critical role of NK cells in editing adaptive immune responses against HIV-1 [14]. In addition, initial HIV-1 eradication studies have shown that NK-cell responses are the major correlates of the decrease in viral DNA levels in a subgroup of ART-treated patients [15,16]. Moreover, a recent study showed that IFN-γ production and activating receptor upmodulation by ex vivo stimulated NK cells were inversely correlated with HIV-1 DNA loads in patients, spontaneously controlling viral replication, indicating that NK cells with a specific functional profile may contribute to HIV-1 reservoir containment [17]. Additional evidence encouraging the exploitation of NK cells in HIV-1 eradication strategies is the finding that, in a simian model of non-pathogenic simian immunodeficiency virus (SIV) infection, NK cells accumulated in secondary lymphoid organs, the major anatomic site of HIV-1/SIV reservoirs, where they exerted efficient control of viral replication [18].

Work performed by various groups including our own has demonstrated that HIV-1-infected T cells are exposed to NK cell killing due to virus-induced upregulation of ligands for the NKG2D receptor (NKG2DLs) [19,20,21,22,23]. More recently, we showed that NKG2DLs upmodulation also occurs on latently HIV-1-infected CD4^+^ T cells once the virus is reactivated [24,25]. The NKG2D receptor is expressed by NK cells as well as by CD8^+^ T cells, subsets of γδ T cells and NKT cells, and recognizes various cell-surface molecules, specifically MHC-class-I-related sequence A (MICA) and B (MICB) proteins and six different cytomegalovirus UL16-binding proteins (ULBP1-6); these NKG2DLs, normally, are not expressed by healthy cells but can be induced in virus-infected or transformed cells [26]. Ligand engagement by NKG2D delivers a potent activating signal to NK cells, in some instances overcoming signals delivered by inhibitory receptors, and a co-stimulus to CD8^+^ T cells, thus functioning as a key activating pathway for the elimination of infected cells and tumors by cytotoxic lymphocytes [27,28,29,30,31,32]. Strengthening the role of NKG2D in anti-HIV-1 NK cell responses, it was shown that NKG2D engagement co-stimulates CD16 signaling, hence enhancing ADCC-mediated killing of HIV-1-infected cells coated with antibody recognizing the viral envelope [33].

Interestingly, several LRAs, such as inhibitors of histone deacetylases (HDACis) or DNA methyltransferases (DNMTis), that have already entered into clinical trials based on their anti-cancer activity, can induce upregulation of NKG2DLs and other NK cell activating ligands on ex vivo treated tumor cells, hence promoting their recognition and elimination by NK cells [34,35,36,37]. Therefore, it is plausible that in CD4^+^ T cells harboring LRA-reactivated HIV-1 the drug cooperates with the virus at upregulating NKG2DLs and sensitizing for NK cell-mediated clearance. This model is supported by recent experimental evidence that will be described here, together with in vivo and ex vivo data showing the potential impact of LRAs on the NKG2D expression and overall cytotoxicity of NK-cells. Finally, here we highlight opportunities to harness the NKG2D pathway towards the goal of HIV-1 eradication in the context of recent advances in NK cell-based immunotherapies.

## 2. Mechanisms Maintaining HIV-1 Latency Can Be Counteracted by LRAs

HIV-1 preferentially infects activated CD4^+^ T cells in which it generally establishes a highly productive replication cycle leading to cell destruction within days. However, in a small number of cells, mainly resting central memory CD4^+^ T cells, the virus establishes a non-productive infection and remains stably integrated in the host genome in a transcriptionally silent form unaffected by ART regimens. Several molecular mechanisms are involved in the establishment and maintenance of HIV-1 latency for which an elaborate overview is given elsewhere [38,39]. Briefly, in the majority of latently infected cells, HIV-1 infection is repressed at the transcriptional levels due to various possible elements: (i) The absence of crucial transcription factors, such as NF-κB and NFAT, that are sequestered in the cytoplasm of resting cells; (ii) the chromatin environment and mechanisms of transcriptional interference at the site of integration into the host cell genome; (iii) the epigenetic control of the HIV-1 promoter mediated by histone acetylation and by methylation of histones or DNA; (iv) the presence of cellular transcriptional repressors, such as Negative Elongator Factor (NELF) and the TRIM family of proteins, and retrovirus-silencing microRNAs; (v) the sequestration of positive transcription elongator factor b (P-TEFb) in an inactive complex; and (vi) the low concentration of the Tat viral protein, which promotes HIV-1 transcription by recruitment at the viral promoter of P-TEFb and chromatin-remodeling factors.

On the basis of this knowledge, a plethora of LRAs belonging to different functional categories have been developed, as recently reviewed elsewhere [40,41,42].

A major category of LRAs consists in compounds that modulate histone post-translational modification, including HDACis and inhibitors of histone methyltransferase (HMTis). By modifying histones within the nucleosomes of integrated HIV-1 genome, these compounds favor accessibility of transcription factors and RNA polymerizing machinery to the viral long terminal repeat (LTR) promoter sequence. Prominent HDACi examples are Suberoylanilide hydroxamic acid (SAHA or Vorinostat), Romidepsin, and Panobinostat, which have already entered into clinical trials showing some HIV-1 reactivation activity in vivo [3].

A distinct functional category of LRAs comprises compounds modulating non-histone elements within the chromatin, such as inhibitors of repressive bromodomain and extraterminal domain proteins (referred to as BETi; e.g., JQ1 and hexamethylene bisacetamide -HMBA) and DNMTi (e.g., 5-aza-2′ deoxycytidine or AZA-CdR).

A remarkable group of LRAs is formed by NF-κB stimulators, mainly consisting of protein kinase C pathway agonists (PKCas) such as Prostratin, Bryostatin, and Ingenol-B, and in the SMAC mimetic class of compounds that activate the non-canonical NF-κB pathway. In general, PKCas are considered the most potent LRAs, yet concerns about their use have been raised as they can cause systemic inflammation. Possibly due to a cautious low-dose administration, Bryostatin had no effect on either PKC activation or HIV-1 reactivation in a pilot clinical trial in ART patients [43], thus new trials assessing higher doses or multiple administrations of Bryostatin could be considered.

Another promising family of LARs is the one of Toll-like receptor agonists (TLRas), which can reactivate latent HIV-1 acting on a variety of pathways also involving non-T cell responses and, at the same time, are capable of stimulating antiviral immune responses. The effects of the MGN1703 TLR-9 agonist have been investigated in a clinical study in ART patients, showing HIV-1 reactivation and stimulation of host immune cells including NK cells [44], while the GS-9620 TLR7 agonists are currently being tested in a phase 1 trial.

Some LRAs do not fit in the above categories and have unique features, such as Disulfiram, a drug used to treat alcoholism that was shown by in vitro studies to stimulate proviral transcription; in clinical trials in HIV-1 patients, though, Disulfiram resulted in a modest reactivation of the latent virus [45].

Other approaches to target the HIV-1 reservoir include administration of proteasome inhibitors displaying LRA capacity (e.g., Bortezomib and Ixazomib) or compounds stimulating cell-surface receptors such as TNF-α, IL-15, and the ALT-803 superagonist of IL-15, this latter molecule being tested for tolerability and efficacy in an ongoing trial in ART patients.

At present, over 160 LRAs have been described and novel drugs with LRA property that have improved efficacy and safety profiles are continuously being developed. Major efforts are devoted to find compounds capable of targeting, more specifically, HIV-1 reactivation without inducing global changes in the host transcriptome, which can be caused by most HDACis and BETis, and devoid of potential adverse immune activity, which was described for some HDACis and PKCas [42].

Moreover, based on the results of initial clinical trials, it seems unlikely that administering a single LRA will be sufficient to eradicate HIV-1. Therefore, combinations of two LRAs belonging to distinct functional categories, such as coupling a PKCa with a HDACi, are under investigation and may enter in future clinical trials depending on their pharmacological suitability [40,42].

## 3. LRAs Can Affect NKG2DLs Expression

The eight NKG2DLs are regulated mostly independently in a very complex manner at both transcriptional and post-transcriptional levels that has been reviewed in detail [26,46,47]. In brief, transcription of *NKG2DL* genes depends on the initiation of the DNA Damage Response (DDR) pathway, chromatin remodeling, and recruitment of activated NF-κB and other transcription factors at their promoter sequences. Translation of NKG2DL mRNAs can be inhibited by several microRNAs and RNA binding proteins. At the protein level, NKG2DLs are regulated through various mechanisms including secondary modifications, intracellular localization, stability, and extracellular release in a soluble form (sNKG2DLs) via proteolytic cleavage or via exosomes (a process called shedding). In normal tissues, NKG2DLs expression is highly restricted but it can be induced following a cell stress such as viral infection and tumor transformation [26]. This stress response, however, is usually contrasted by immune evasion mechanisms developed by both viruses and cancer, such as NKG2DL mRNA degradation and protein intracellular retention or shedding in soluble form. Numerous drugs playing an important role in the treatment of cancer patients share the ability to upregulate NKG2DLs in transformed cells, hence are capable to sensitize tumors to NKG2D-mediated recognition and killing by NK cells [34,35,36,37]. Of note, several of these anticancer drugs are currently under investigation for the employment in “shock-and-kill” strategies based on their capacity to reactivate latent HIV-1. Among various potential immunomodulatory mechanisms acting on NK-cell targets as well as directly on NK cells, the NKG2DL upregulation activity is shared by several drugs for which both anticancer and LRA properties have been reported. Specifically, candidate LRAs that were shown to enhance NKG2DL expression on in vitro-exposed cancer cell lines and primary tumor cells include several HDACis (Valproic acid, Trichostatin A, Sodium Butyrate, Romidepsin, Panobinostat, and SAHA), proteasome inhibitors (MG132 and Bortezomib), DNMTi (AZA-CdR), and BETi (JQ1) [36,37]. On the basis of this evidence, we recently proposed a model for which latent HIV-1 and NKG2DLs are under the control of common regulatory mechanisms and provided experimental data (described here below) showing that it is possible to select drugs for HIV-1 eradication strategies that are efficacious at reactivating the latent provirus while, at the same time, effectively enhancing NKG2DL expression on the membrane of T cells that exit from latency [24,25].

## 4. HIV-1 Affects NK Cell Recognition by Modulating NKG2DLs

To escape from recognition by cytotoxic lymphocytes, HIV-1 has evolved a multifaceted strategy acting at various levels. One key immune evasion mechanism is exerted by the Nef viral protein that specifically binds and downregulates HLA-A and -B molecules but leaves unaffected HLA-C and -E expression, resulting in impaired recognition and killing of infected cells by HIV-specific CD8^+^ T cells and, simultaneously, in the protection from NK cell responses, at least of those NK cells expressing inhibitory receptors specific for HLA-C or -E [48]. This model was refined by later studies showing that in most primary HIV-1 isolates, the viral Vpu protein has evolved the capacity to downmodulate HLA-C to variable degrees [49], so that HLA-C-licensed NK cells can kill HIV-1-infected cells in a manner that depends on the strength of KIR/HLA-C haplotype interaction and on the extent of virus-mediated HLA-C downregulation [50].

Furthermore, in line with the crucial role of NKG2D-mediated responses in NK cell antiviral function, HIV-1 has developed various strategies interacting with the NKG2D/NKG2DL axis. On one hand, in HIV-1-infected CD4^+^ T lymphocytes, transcription of *NKG2DL* genes (*MICA*, *MICB*, and *ULBP1-2*) is induced by the viral Vpr protein via activation of the DDR pathway, resulting in increased ligand expression at the cell membrane [19,20,21,22,23,51,52]. Specifically, the stimulatory effect of Vpr on NKG2DLs expression is due to the protein ability to interact with the cellular cullin-ring E3 ubiquitin ligase (DDB1-CUL4A) and to activate the ATR DNA damage sensor kinase, a key regulator of the DDR pathway [21,53]. The same Vpr interactions that are required for HIV-1-infected cells to arrest in the G_2_ phase of the cell cycle allows efficient production of viral particles [54], hence it is plausible that NKG2DLs upregulation is a ‘side effect’ of an HIV-1 function essential for viral replication. Analogously, Vpr also induces PVR, a ligand for the DNAM-1 activating receptor of NK cells, although acting at a different post-transcriptional level not yet identified [55]. In HIV-1-infected cells, additional factors may sustain the DDR pathway and, consequently, NKG2DLs upmodulation, such as the activation of the ATM DNA damage sensor kinase during viral DNA integration [56] and the triggering of the apolipoprotein B-editing complex 3G (APOBEC3G), a cellular antiviral factor that deaminates cytidine residues [57]. On the other hand, NKG2DLs upmodulation is counterbalanced by opposite HIV-1 activities that reduces cell-surface ligand expression. Work from our group has demonstrated that the HIV-1 Nef protein reduces the cell-surface expression of MICA, ULBP1, and, especially, ULBP2, thus impairing NKG2D-dependent killing by NK cells [19]. In the infected cell, Nef acts as a versatile adaptor that removes several resident proteins from the cell membrane, such as HLA-A/B and CD4, through increased protein internalization, retention in intracellular compartments, or re-directing to lysosomes for degradation [58], which has important functional consequences for viral replication [59]; nevertheless, based on mutational analysis, Nef uses distinct mechanisms to affect cell-surface NKG2DL expression that have not yet been identified [19]. The importance of NKG2DL downregulation by Nef in HIV-1 pathogenesis is strongly suggested by the fact that this activity is conserved in Nef protein variants derived from patients with chronic infection [19,60], with the exception of elite controllers (ECs), patients who spontaneously suppress HIV-1 in the absence of ART [61]; importantly, Nef variants from EC patients were shown to enhance the susceptibility of HIV-1-infected T cells to ADCC via increased NKG2D/NKG2DLs interaction [61], confirming previous evidence that NKG2D acts as a co-receptor for anti-HIV-1 ADCC [33]. Adding to the downmodulation activity mediated by Nef, another HIV-1 protein, Vif, contrasts NKG2DL expression indirectly, through its capacity to target APOBEC3G to degradation dampening DDR pathway activation [57].

Despite virus countermeasures, T cells infected in vitro with HIV-1 express higher NKG2DLs levels as compared to non-infected cells and are killed by NK cells through NKG2D activation [19,20,51,57,62]. Of note, ex-vivo activated HIV-1-infected CD4^+^ T cells derived from patients upregulate ULBP2 and are killed by autologous NK cells in a NKG2D-dependent manner [20].

It should be mentioned that NKG2DLs are not the only NK cell activating ligands where their expression is affected by HIV-1; indeed, Nef also inhibits expression of the ligand for the NKp44 activating receptor [63] and of the PVR ligand for DNAM-1, assisted in this latter activity by Vpu [62]. Moreover, Vpu reduces NK cell degranulation by downregulating NTB-A on the surface of infected cells [64]. Yet, blocking DNAM-1 or NTB-A with specific antibodies reduces NK cell-mediated lysis of HIV-1-infected cells, even though to a modest extent, suggesting that residual expression of ligands for these receptors is sufficient for delivering an activating signal to NK cells. Overall, experimental evidence based on the use of receptor blocking antibody in cytotoxicity assays, has demonstrated that NK-cell mediated killing of HIV-1-infected cells mainly occurs via NKG2D/NKG2DL interaction, but is enhanced when DNAM-1 or NTB-A co-activating receptors are simultaneously engaged [62,64]. Further investigations could possibly reveal that other activating receptors act synergistically in mediating NK cell reactivity against HIV-1-infected targets [30].

Finally, work performed in the laboratory of M. Altfeld and G. Alter and by our group has shown that in vitro HIV-1-infected CD4^+^ T cells release sNKG2DLs via the proteolytic activity of matrix metalloproteinases (MMPs) [23,65], cellular enzymes that are found upmodulated in chronic HIV-1 infection [66]; this in vitro evidence was corroborated by the observed accumulation of soluble MICA (sMICA) in the plasma of viremic HIV-1-infected patients [23,65].

Shedding of sNKG2DL is a phenomenon well characterized in cancer patients in whom high plasma levels of sNKG2DL released by transformed cells can not only decrease cell-surface ligand expression, but also inactivate/downmodulate NKG2D on circulating lymphocytes and allow efficient evasion of NKG2D-mediated anti-tumor responses [67]. On the other hand, shed MULT1, a murine ULBP1 ortholog with high affinity for NKG2D, competed with tumor-bound ligands for receptor engagement, thus reversing NKG2D desensitization and promoting tumor rejection in a mouse model [68]. Therefore, as yet-obscure molecular mechanisms behind the shedding of sNKG2DLs and implications for HIV-1 pathogenesis deserve further investigation, as discussed in a recent review [69].

## 5. Latent HIV-1 and NKG2DLs Are Regulated via Common Mechanisms That Can Be Modulated by LRAs

Since information on NKG2DLs expression in the context of HIV-1 reactivation was missing, we used an experimental primary CD4^+^ T-cell model of HIV-1 latency to measure expression of MICA, MICB, and ULBP2 upon virus reactivation with SAHA or, as maximal stimulation, with PMA and Ionomycin. Our results showed that both stimuli upregulated ULBP2 and, to a lesser degree, MICA/B on p24^+^ (Gag capsid antigen) cells harboring reactivated virus as compared to p24^−^ cells in the same stimulated cultures or to spontaneously reactivated p24^+^ cells in non-stimulated control cultures, indicating that enhanced NKG2DL expression was due to HIV-1 reactivation in the presence SAHA or PMA/Ionomycin, while spontaneous HIV-1 reactivation or drugs alone were not sufficient to induce this phenomenon [24]. Moreover, when latency was established with a Nef-deficient virus, NKG2DLs were further upmodulated on SAHA-reactivated p24^+^ cells, confirming that Nef negatively affected ligand expression also in the context of latent HIV-1 reactivation. These data provided a proof of principle that in primary CD4^+^ T cells NKG2DLs are induced by integrated HIV-1 upon reversal of latency. We further investigated this phenomenon using latently HIV-1-infected T cell lines (J-Lat 6.3/8.4/9.2 and J1.1) in which expression of NKG2DLs was analyzed at both mRNA and protein levels following stimulation with SAHA in the presence or absence of ATM/ATR inhibitors. Our results indicated that both SAHA and reactivated HIV-1 stimulated expression of MICA, MICB, and ULBP2 (but not ULBP1) with an additive effect; yet, some viral factor (likely Nef) mediated intracellular ligand retention, especially of MICA and ULBP2 proteins. Nevertheless, despite that only a modest ligand upmodulation was induced by SAHA on p24^+^ if compared with p24^−^ cells, SAHA potently enhanced NK cell-mediated killing of T cell targets with a stronger effect against p24^+^ cells. This NK cell-mediated lysis was dependent on NKG2D, since it was inhibited by a anti-NKG2D blocking antibody and, in addition, it could be potently enhanced by pre-exposing NK cells to IL-2, IL-7, and, especially, IL-15 in a manner that was directly correlated with the level of cytokine-induced NKG2D upmodulation. Interestingly, the activity of the ATR kinase was required for NKG2DL upregulation as well as for efficient HIV-1 latency reversal activities of SAHA, which is in line with previously reported data showing that ATR/ATM kinases contribute to HDACi-induced expression of NKG2DLs [70] and that ATR-mediated G_2_ cell cycle arrest by the viral Vpr protein is needed for efficient HIV-1 expression [71]. Since SAHA, as all HDACis, can target histones as well as a variety of non-histone proteins [72], cellular factors controlling both latent HIV-1 and NKG2DL expression other than ATR could be modulated by this drug. One candidate is Sp1, a transcription factor that is recruited at the HIV-1 LTR following SAHA-induced histone modifications and chromatin remodeling [73,74]; Sp1 binding sites are present also in NKG2DL promoter regions [75], hence it would be interesting to test whether Sp1 activates transcription of *NKG2DL* genes in SAHA-treated cells. In addition, SAHA-induced activation of the PI3K/Akt pathway has been implicated in increased transcription of both HIV-1, via recruitment of P-TEFb to the viral LTR [76], and *MICA/B* genes, through the activity of the downstream GSK-3 kinase [77].

In a second study, we analyzed the impact on NKG2DLs expression upon viral reactivation with a PKCa, Prostratin or Bryostatin, in a primary CD4^+^ T-cell model of HIV-1 latency [25]. The result showed that ULBP2 expression was increased by both drugs in T cells with or without HIV-1 reactivation, yet only Prostratin upregulated ULBP2 to a significantly higher level on p24^+^ cells if compared with p24^−^ cells in the same cultures or with spontaneously reactivated p24^+^ cells in non-stimulated cultures. In parallel, ULBP2 mRNA was quantified showing that, while HIV-1 latent infection by itself resulted in a slight increase of ULBP2 mRNA levels, the sole exposure to Prostratin or Bryostatin led to a ULBP2 mRNA increase by 16- or 10-fold, respectively; most importantly, ULBP2 mRNA was further increased, up to 43-fold, when Prostratin, but not Bryostatin, was added to latently infected T cells. Therefore, while both drugs can upregulate ULBP2 at the transcriptional level, only Prostratin synergized with reactivated HIV-1 at upregulating ULBP2. Alongside, clearance of T cells that exit viral latency by autologous NK cells was increased when both targets and effectors were exposed to Prostratin but not to Bryostatin. This latter result could be influenced by the differential effect that the two PKCas can have on the cytotoxic function of NK cells (discussed here below). Of note, by means of blocking antibody, we could show that the NKG2D receptor mediated killing of p24^+^ T cells by NK cells in Prostratin-exposed co-cultures. Activation of the PKC pathways by Prostratin and Bryostatin may occur via stimulation of different enzyme isoforms, ultimately resulting in the activation of NF-κB that is a central mediator of latent HIV-1 expression [78,79] as well as a transactivator of *NKG2DL* genes [26]. Therefore, further work is required to decipher the modality of PKC activation used by the two drugs and through which mechanisms Prostratin, but not Bryostatin, synergizes with HIV-1 at upregulating ULBP-2 expression.

Overall, we provided in vitro evidence that some LRAs can simultaneously revert viral latency and upregulate NKG2DL expression, eliciting NK cell-mediated clearance of cells harboring reactivated HIV-1. Our work, together with a number of studies referenced herein, clearly indicates that latent HIV-1 and NKG2DLs are under the control of common epigenetic mechanisms, transcription factors, and regulatory pathways, including DDR, PI3K/Akt, and PKC pathways, which can be regulated by LRAs resulting in de-repressed/increased expression.

## 6. Impact of LRAs on NK Cell Phenotype and Function

A matter of concern in the design of ‘shock and kill’ intervention is the possibility that selected LRAs may exert deleterious effects on the host immune effectors cells. In fact, ex vivo exposure of CTLs to various HDACis or PKCas was shown to suppress different aspects of their biology and, most importantly, to inhibit CTL cytotoxicity against HIV-infected T cells [80,81,82]. Analogous studies were performed on NK cells, showing a negative impact of SAHA, Romidepsin, and Panobinostat on NK cell viability and/or cytotoxic activity [83,84,85,86]. When expression of NK cell activating receptors was analyzed, various HDACis showed diverse negative effects, the worse being observed with Panobinostat that downregulated all activating receptors including NKG2D [84]. Recently, we showed that ex vivo NK cell exposure to a BETi compound, HMBA, reduced expression of NKG2D and its DAP10 adaptor molecule, impairing NKG2D-mediated cytotoxicity and dampening NK cell capacity to respond to IL-15 stimulation which is dependent on DAP10 [87]. Conversely, work performed by different groups including ours have shown that exposure to Prostratin results in overall activation of NK cells associated with upmodulation of NKG2D and NKp44 receptors, shedding of CD16, and, most importantly, in an enhanced NK cell-mediated clearance of T cells latently or productively infected with HIV-1 [25,84,85]. Of note, we were able to show that Prostratin-treated NK cells cleared autologous HIV-1^+^ cells reactivated by the same drug and that this killing was in part dependent on NKG2D engagement [25]. On the other hand, this was not the case for Bryostatin, a PKCa that shared with Prostatin the capacity to activate NK cells; most importantly, the natural cytotoxicity and ADCC function of NK cells were inhibited by Bryostatin in most donors, while both activities were either improved or maintained by Prostratin. Future studies will possibly identify other PKCas endowed with both LRA and NK-cell stimulatory activity; a good candidate is Gnidimacrin, a potent PKCa that was shown to eliminate latent HIV-1 in a CD8^+^ T cell-independent manner in ex vivo exposed PBMCs of ART patients [88]. Furthermore, Bortezomib enhanced the capacity of infused NK cells to clear a NKG2DL^+^ tumor in mice [89]; therefore, the possibility that proteasome inhibitors can stimulate in vivo NKG2D-mediated NK cell responses should be considered. Finally, it will be interesting to test whether TLRas, that function simultaneously as LRAs and immunomodulatory compounds, may induce clearance of reactivated HIV-1^+^ cells via NKG2D-mediated NK cell cytotoxicity.

Overall, ex vivo studies give useful preliminary information on LRA applicability in HIV-1 eradication strategies. However, it should be emphasized the importance of confirming results in vivo, since ex vivo data do not fully reflect conditions found in patients. Actually, the reported inhibitory effects of SAHA and Panobinostat on ex vivo exposed NK cells have been ultimately contradicted by functional tests on NK cells isolated from ART patients treated with these drugs [15,16].

## 7. NK Cell Response in HIV-1-Infected Patient on ART

HIV-1 causes pathologic changes in NK cell frequencies, phenotype, and function that are initiated in the acute phase of infection and persist as disease progress towards the chronic phase. These events have been extensively reviewed elsewhere [90,91] and, in brief, consist in progressive decline of circulating cytotoxic CD56^dim^ NK cells replaced by an expanded dysfunctional CD56^neg^ subset expressing high levels of inhibitory receptors and expression of activating receptors and characterized by reduced killing, ADCC, and cytokine production activities. The major cause of CD56^neg^ cells expansion is attributed to prolonged NK cell activation by chronic exposure to HIV-1-infected cells, although other mechanisms should not be excluded. As to NKG2D, decreased expression of this receptor on NK cells of viremic untreated patients was associated with high plasma levels of circulating sNKG2DL, especially sMICA, released by infected T cells, a defect that could impair NK cell responses against HIV-1 as well as other opportunistic infections or cancer [23,65,69].

Importantly, ART uptake and consequent suppression HIV-1 viremia largely restores the NK cell compartment in terms of reconstitution of normal subsets distribution, phenotype, and function of NK cells [90]. However, studies on ART patients have produced some diverging and contradictory results, so that there is no consensus on the degree, either partial or complete, to which the phenotype and function of NK cells are actually reset to normal levels in treated patients. This controversy is likely related to multiple factors: Many studies are cross sectional and may not be adequately controlled for confounders such as timing of ART initiation, co-infections status, or age; finally, selected NK cell markers may have not fully captured relevant cell subsets.

Also controversial is the expression of NKG2D on NK cells of patients on virus suppressive ART: NKG2D levels were comparable to those observed on NK cells of healthy subjects in some studies [23,92], while they were relatively decreased [65] or, conversely, increased [93] in other studies. Furthermore, as compared to healthy subjects, the expression of NKG2D on NK cells of EC patients was similar in one study [65], but higher [17,93] or lower [94] in other reports. These results suggest that HIV-1-independent variables can influence NKG2D expression levels, such as individual variations and polymorphisms of the NKG2DL/NKG2D system. Overall, several aspects of NKG2DL regulation and their consequences for NKG2D expression/function in both healthy and HIV-1-infected individuals are still elusive [47,69]. On the other hand, more homogenous results can be obtained in longitudinal studies. Indeed, analysis of the same patients before and after ART uptake disclosed that viral suppression resulted in drastic NKG2D increase on NK cells associated with a reduction of circulating sNKG2DLs [23], indicating that ART has the potential to restore NKG2D-mediated responses of NK cells in HIV-1-infected individuals.

## 8. NK Cell Function in HIV-1 Cure Clinical Trials

Although not sufficient for HIV-1 eradication, the potential of NK cells has recently emerged as superior to that of CD8^+^ T cells in completed clinical trials in which Panobinostat or Vorinostat was administered to patients on virus suppressive ART. Both drugs increased cell-associated HIV-1 RNA in CD4^+^ T cells, yet failed to achieve an overall significant reduction of latently infected cells [95,96,97,98]. Interestingly, results from the Panobinostat trial showed that NK-cell responses, not HIV-1-specific CTLs, were the major correlates of the reduction in CD4^+^ T-cell associated HIV-1 DNA observed in a subgroup of treated patients [15]. Specifically, the frequency of total NK cells and of CD56^dim^ NK cells expressing CD57 and CD69 activation markers were inversely correlated with HIV-1 DNA levels throughout the trial. Analogously, a recent study demonstrated that, in ART-treated patients that were administered with multiple SAHA doses, the frequency of NK cells, but not that of CD4^+^ or CD8^+^ T cells, was increased and NK cells displayed a trend towards higher expression of activating receptors, in particular CD16 and NKp46, and enhanced functionality in terms of degranulation and IFN-γ production [16]. In the same study, by analyzing all together various samples of Panobinostat and SAHA trials, the authors found a significant inverse correlation between frequencies of NK cells, (either total or CD16^+^ NK cell frequencies) and HIV-1 DNA levels in CD4^+^ T cells, indicating that enhanced NK cell phenotype and function might have an impact on the viral reservoir size. In addition, a positive correlation was found between NKG2D expression and HIV-1 DNA; this unexpected association is in apparent contrast with the antiviral role of NKG2D and deserves further investigation.

Overall, the role of NK cells in HIV-1 eradication has been investigated in a small number of clinical studies, but this limited information will be improved once various ongoing trials are concluded. On the basis of the current knowledge, it is expected that the capacity of NK cells to clear the HIV-1 reservoir will be stimulated in vivo by therapies including specific interventions that were shown to boost NK cell responses against HIV-1 in several ex vivo and in vivo studies, such as administration of the IL-15/IL-15 superagonist [24,99,100], IFN-α [101,102], and TLR-7/9 agonists [44,103,104]. Interestingly, IL-15 and IFN-α share the capacity to boost NKG2D-mediated NK cell cytotoxicity [24,101,102]; this is also true for a TLR-8 agonist [105] while, to the best of our knowledge, TLR-7/9 agonists have not been tested for this activity, as yet.

## 9. Exploiting NK Cells to Eradicate HIV-1

Results from clinical trials mentioned above encourage further exploration of NK cell-based approaches to achieve HIV-1 eradication. To reach this goal, though, some important matters should be clarified, as discussed here below.

### 9.1. Questions to Be Addressed

(i) Does latent HIV-1 contain NK cell-escape mutations?

Evidence has been provided that latent HIV-1 reservoirs contain mutations resulting in viral peptide variants that can enhance binding of inhibitory NK cell receptors (e.g., KIR2DL) to HLA/peptide complexes leading to reduced antiviral NK cell cytotoxicity [13]. The extent and the relevance of NK cell escape mutations in HIV-1 reservoirs are currently unknown and deserve to be investigated.

(ii) Which NK cell subset is best fit for killing reactivated HIV-1?

According to in vivo studies, some distinct NK cell subsets were accredited with HIV-1 eradication potential. Results of the Panobinostat trial suggested that a NKp46^+^CD56^dim^ subset correlated with most favorable outcome in patients [15]. On the other hand, in patients co-infected with human cytomegalovirus (HCMV) and HIV-1 that were treated with pegylated IFN-α, reduction of the HIV-1 reservoir was attributed to expanded NKG2D^+^NKp30^+^CD56^bright^ NK cells [102]. Although not investigated in HIV-1 eradication studies as yet, a memory-like NKG2C^+^CD57^+^KIR^+^CD56^dim^ NK cell subset, that was identified in HCMV-seropositive individuals and greatly expanded in HIV-1/HCMV co-infected patients, represents a great promise [106]. Indeed, this long-living NK cell subset has adaptive immune traits showing superior ADCC activity and cytotoxicity against cells infected with HIV-1, HCMV, or other viruses when compared to conventional NK cells [107]. In SIV-infected rhesus macaques, the existence of antigen-specific NK cells endowed with robust cytotoxic activity against Gag- and Env-pulsed dendritic cells has been demonstrated [108]. Of note, antigen-specific killing of primate NK cells was shown to occur in an NKG2D-dependent manner. A greater understanding of the biology of human memory-like NK cells is highly needed and it will inform new strategies exploiting their potential in the fight against HIV-1. Finally, a study by Lisovsky et al. demonstrated that NKG2A^+^ NK cells are those that better respond in vitro to autologous HIV-1-infected CD4^+^ T cells by developing antiviral functions (i.e., degranulation, production of IFN-γ, and the CCL4 chemokine) [109]. Both NKG2A and NKG2C receptors recognize HLA-E molecules which present leader peptides derived from other class I molecules, but they deliver opposite signals, inhibitory and activating, respectively, and are usually expressed on distinct NK cell populations [110]. In their study, Lisovsky and colleagues suggested that peptides derived from HLA-A/B molecules downregulated by Nef as well as HIV-1-encoded peptides may stabilize HLA-E on the surface of infected cells hence favoring recruitment of NKG2A^+^ NK cells that will be ultimately activated via triggering of activating receptor/ligands interactions, although these latter were not analyzed [109]. Overall, further investigation is needed in order to elucidate which specific NK cell subset should be potentiated to achieve HIV-1 clearance.

(iii) Which is the preferable approach to an NK cell-based strategy for HIV-1 eradication?

It is hoped that results from ongoing clinical trials will demonstrate that specific interventions can reactivate HIV-1 and induce immune-mediated reduction of the viral reservoir size in ART patients. Some of these interventions, such as the administration of TLRas or the combination of Panobinostat with pegylated IFN-α, have the potential to activate host antiviral NK cell responses, at least on the basis of previous ex vivo and clinical studies. Alternatively, or in addition, boosting NK cell function via ad hoc strategies represents a useful adjuvant strategy in a multipronged approach to HIV-1 eradication. Enhancement of NK cells, for instance, could be highly beneficial for patients receiving bNabs that are able to neutralize multiple HIV-1 strains through direct virus interaction and, to a large extent, by means of NK cell-mediated ADCC [111]. In this matter, recent advances in the understanding of NK cell biology and translational applications have paved the way for innovative therapies that are being tested in cancer patients and already showed signs of efficacy in various clinical trials [112]. In particular, adoptive transfer of ex vivo-expanded allogeneic NK cells (i.e., isolated from PBMCs or generated from CD34^+^ progenitor cells of an unrelated donor) was safe and efficacious in therapeutic trials against hematological malignancies, although relatively ineffective against solid tumors [113]. Moving forward in this field, many research laboratories and biotech companies are developing a plethora of approaches to augment the function of NK cells, especially if combined with NK cell adoptive transfer therapy; these approaches include systemic administration of NK cell enhancing products (e.g., IL-15 or its ALT-803 superagonist, antibodies blocking NK cell inhibitory receptors, monoclonal antibodies-mAbs-with enhanced binding affinity to CD16) and genetic modification of NK cells (e.g., chimeric antigen receptor-CAR-engineered, overexpression of IL-15 or homing molecules) [113,114,115].

Results from several ongoing clinical trials are eagerly awaited as they may have a considerable impact on cancer patients and also bring novel therapeutic opportunities for different clinical settings including HIV-1 infection.

### 9.2. NKG2D-Based Therapy: Lessons from Cancer Studies

**Figure 1:**
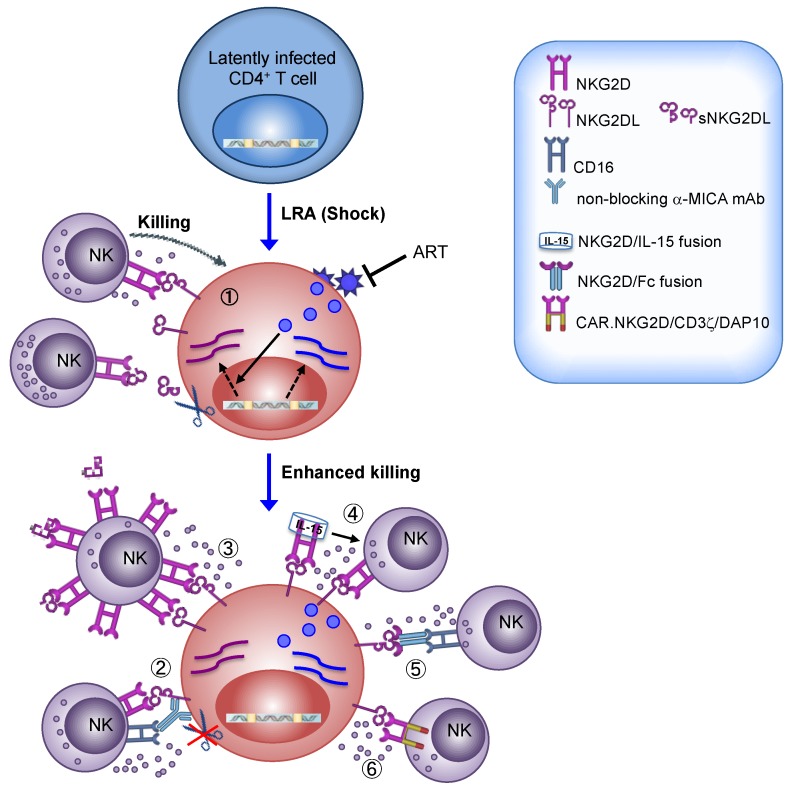
Diverse approaches targeting the NKG2D/NKG2DLs axis for ‘shock and kill’ HIV-1 eradication strategy. (**1**) The ‘shock’ phase relies on the use of LRAs to increase HIV-1 transcription within latently infected CD4^+^ T cells while ART blocks the spread of newly assembled viral particles. The expression of NKG2DLs is induced by the Vpr and Vif viral proteins and, possibly, by other virus-induced mechanisms. Several LRAs can induce the expression of NKG2DLs and these drugs may cooperate with HIV-1 at NKG2DL upmodulation, sensitizing T cells that exit from latency to recognition and killing by NK cells. On the other hand, due to proteolytic shedding by activated MMPs, sNKG2DL can be released in the extracellular milieu, bind and downregulate NKG2D on NK cells. (2–6) Various approaches to boost the NKG2D-mediated responses of NK cells have been developed and could be applied as an adjuvant NK-cell based immunotherapy to clear T cells harboring reactivated HIV-1: (**2**) Treatment with an anti-MICA mAb that blocks shedding but not NKG2D recognition resulted in enhanced NK cell-mediated killing by triggering both NKG2D pathway and ADCC [117,118]; (**3**) adoptive transfer of IL-2-activated NK cells expressing high NKG2D levels reduced the amount of plasma sNKG2DL and improved clearance of NKG2DL^+^ targets [119]; (**4**) recombinant NKG2D fused to IL-15 activated NK cell-mediated suppression of NKG2DL^+^ cells [120,121]; (**5**) the extracellular domain of NKG2D fused to the Fc of IgG_1_ optimized for CD16 binding elicits ADCC against NKG2DL^+^ targets [122,123]; (**6**) CAR-NK cells engineered to express a NKG2D/CD3ζ/DAP10 recombinant receptor display superior NKG2D-mediated cytotoxic activity [124,125]. LRA, latency-reversing agent; ART, antiretroviral therapy; NKG2DL, NKG2D ligand; sNKG2DL, soluble NKG2D ligand; MMP, matrix methalloprotease; mAb, monoclonal antibody; ADCC, antibody-dependent cellular cytotoxicity; Fc, fragment crystallizable region; CAR, chimeric antigen receptor. Dotted black arrow represents gene expression; solid black arrow represents stimulation.

The expression of NKG2DLs on tumors cells of diverse origin has made targeting the NKG2D/NKG2DLs axis an attractive therapy for the treatment of many cancer types [46,116]. Several approaches that have been undertaken to harness NKG2D-mediated responses of NK cells against cancer could be applied to HIV-1 eradication strategy (overviewed in Figure 1).

First of all, many anticancer drugs with LRA properties are able to upmodulate NKG2DLs in tumor cells hence promoting their recognition and killing by NK cells [34,35,36,37]. As already described herein, we demonstrated that SAHA and Prostratin cooperate with HIV-1 by increasing NKG2DL expression on the surface of T cells that exit from viral latency, sensitizing to NKG2D-mediated killing by NK cells [24,25] (Figure 1). Based on these preliminary results obtained in vitro, we suggest that ART patients can benefit more from “shock and kill” regimens if using LRAs that not solely reactivate latent HIV-1 but also efficiently enhance NKG2DLs expression and, consequently, NKG2D-mediated immune surveillances. In this matter, important information is provided by the extensive research performed on LRAs that have been already approved as anticancer drugs for the treatment of different malignancies and that have entered into clinical trials [35,36]. Indeed, these studies underscored that some but not all anticancer/LRA drugs may have unwanted side effects such as immunosuppressive activity on NKG2D-mediated responses. One critical aspect is the potential release of sNKG2DL, a phenomenon that may be induced by those HDACis augmenting MMP expression [126] but, conversely, may be inhibited by other LRAs, such as AZA-CdR [127].

High plasma levels of soluble NKG2DLs associated with downregulation of NKG2D on circulating NK cells were found in patients with untreated HIV-1 infection [23,65] or with diverse malignancies [128]; as accumulation of sNKG2DL in the plasma of patients with various tumors correlates with disease progression, metastasis, and poor survival, several approaches have been undertaken to inhibit sNKG2DL shedding that could be of use also in the context of a LRA therapy in ART patients. Very promising results were obtained in humanized mouse models in which treatment with antibodies that target the site of proteolytic shedding of MIC proteins without affecting their binding to NKG2D resulted in impairment of MIC release from the surface of human tumor cells and, most importantly, in tumor elimination mediated by NK cells through activation of both NKG2D and CD16 [117,118] (Figure 1). Another promising approach was developed in neuroblastoma patients infused with high doses of IL-2-activated allogeneic NK cells displaying elevated NKG2D levels; results from this trial showed that the treatment reduced sMICA plasma levels and improved NK-cell cytotoxicity, indicating that NKG2D overexpression can quantitatively scavenge soluble ligands and preserve cytotoxicity of NK cells via non-occupied NKG2D [119] (Figure 1).

As a general approach against virtually all forms of cancer, the combination of NK cell adoptive transfer with anticancer drugs that restore expression of NK cell activating ligands has been proposed as a novel strategy for cancer immunotherapy [36]. Hence, we speculate that a combinatorial approach of LRAs acting on NKG2DLs with adoptive transfer of activated NK cells expressing high NKG2D levels may represent an innovative “shock and kill” strategy against HIV-1 (Figure 1). This may also invigorate the full anti-HIV-1 potential of NK cells; indeed, NKG2D triggering has the potency to enhance the overall cytotoxicity of NK cells by reducing the activation threshold for various NK-cell activating receptors including NKp46, 2B4, and CD16 [30,129]; more specifically, NKG2D stimulation was shown to promote CD16 signaling enhancing ADCC responses of NK cells against HIV-1-infected T cell targets [33].

Furthermore, other innovative strategies have been developed by using the extracellular NKG2D domain fused to immune activating components, such as constant IgG domain that, via optimized CD16 engagement, elicits ex vivo ADCC against NKG2DL^+^ leukemia or breast cancer targets [122,123], and IL-15, which results in activation NK cell-mediated suppression of NKG2DL^+^ human tumors in humanized mice models [120,121] (Figure 1).

Finally, future directions also include the transfer of CAR.NKG2D-engineered NK cells. In particular, NK cells expressing a chimeric NKG2D receptor containing DAP10 and CD3ζ signaling domains were armed with superior cytotoxic activity against human tumors both in vitro and in immunodeficient mice [124,125] (Figure 1). Actually, CAR-NK therapy is potentially safer and more potent than CAR-T therapy, hence it has recently witnessed increasing interest [115].

Overall, several approaches targeting the NKG2D/NKG2DLs axis have been undertaken with some being under clinical evaluation and holding promise of becoming powerful immunotherapies.

## 10. Conclusions and Perspectives

At present, despite extensive efforts to develop an efficacious ‘shock and kill’ approach, an HIV-1 cure is still elusive. Herein we reviewed experimental and clinical evidence suggesting that NKG2D-mediated responses of NK cells have an important role during the ‘killing’ phase and that boosting the NKG2D/NKG2DLs axis is an attractive adjuvant approach to HIV-1 eradication. However, to deliver an effective and tailored NKG2D-based therapy to HIV-1-infected patients, several questions remain to be addressed. A crucial step consists in identifying which LAR (or 2-LRAs combination) could effectively reactivate latent HIV-1 and simultaneously induced high NKG2DL expression, hence opening a ‘window of opportunity’ for NKG2D-mediated immune recognition and clearance of reactivated viral reservoirs; an ideal LRA should also be devoid of any immune suppressive effect on NK cells or other effector cells, either direct (inhibition of activating pathways) or indirect (e.g., via sNKG2DL release). Moreover, further investigation is warranted to identify a strategy to harness the antiviral NKG2D-mediated responses of NK cells in HIV-1-infected patients. Various protocols for the expansion and potentiation of NK cells to be adoptively transferred in cancer patients have been designed and several NK-cell based trials are ongoing. The preclinical success of such studies offers great promise for the applicability of these therapeutic approaches to HIV-1 eradication attempts.
